# Individualized finite element simulation integrating high-resolution magnetic resonance imaging and clinical imaging validation elucidates the effects of laminoplasty and laminectomy on postoperative mechanical stability and degeneration risk

**DOI:** 10.3389/fbioe.2026.1763758

**Published:** 2026-07-14

**Authors:** Yangjing Cao, Duo Shan, Feng Pan, Jinlong Li, Guoli Wang, Yan Sun

**Affiliations:** 1 2nd Department of Orthopedics, Tianjin Jinnan Hospital, Jinnan Hospital, Tianjin University, Tianjin, China; 2 Department of Orthopedics, Tianjin Hospital, Tianjin University, Tianjin, China

**Keywords:** adjacent segment degeneration, finite element modeling, laminectomy, laminoplasty, magnetic resonance imaging-based validation, posterior cervical decompression

## Abstract

**Introduction:**

Posterior cervical decompression is widely used to relieve spinal cord compression; however, postoperative spinal stability and the risk of adjacent segment degeneration (ASD) remain important clinical concerns. Laminoplasty and laminectomy are the two principal posterior decompression procedures and differ substantially in their biomechanical consequences, yet systematic quantitative comparisons are still limited.

**Methods:**

In this study, an individualized three-dimensional finite element (FE) model integrating high-resolution magnetic resonance imaging (MRI) and computed tomography (CT) data was developed to compare these two procedures with respect to postoperative stability, load redistribution, adjacent-segment biomechanical risk, and fixation-related mechanical response. Quasi-static simulations of cervical motion were combined with postoperative MRI evaluation and clinical follow-up data for validation.

**Results:**

The results showed that laminoplasty was more effective than laminectomy in limiting postoperative increases in cervical mobility, reducing intradiscal pressure (IDP), and alleviating facet joint stress, thereby lowering the biomechanical risk of ASD. In addition, an imaging-mechanics coupled degeneration scoring system showed good predictive performance (area under the curve [AUC] = 0.87).

**Discussion:**

These findings clarify the biomechanical mechanisms by which different surgical strategies shape the postoperative mechanical environment and degeneration risk, and provide a quantitative framework to support individualized surgical planning and precision medicine in cervical spine surgery.

## Graphical Abstract

**Figure:**
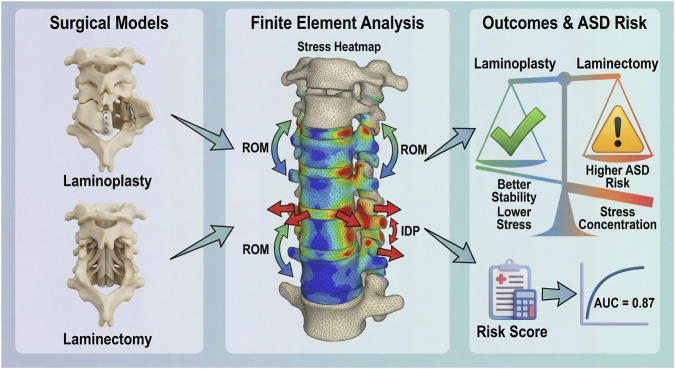
Personalized FE modeling reveals biomechanical differences in postoperative stability and degeneration risk between cervical laminoplasty and laminectomy.

## Highlights

- A precise three-dimensional FE model is constructed from high-resolution MRI and CT data to systematically evaluate biomechanical differences between laminoplasty and laminectomy.
- Laminoplasty demonstrates a marked advantage in reducing the postoperative increase in overall spinal mobility and in optimizing the distribution of LF tension.
- Laminoplasty significantly decreases IDP and facet stress concentration in adjacent segments, thereby reducing the long-term risk of degeneration.
- By integrating imaging and biomechanical outcomes, a postoperative degeneration risk prediction system is developed, showing high diagnostic performance (AUC = 0.87).
- A high-fidelity FEA further characterizes procedure-related differences in implant fatigue performance and stress distribution, providing additional evidence for individualized postoperative management.

## Introduction

Posterior cervical decompression and fixation is a well-established spinal procedure for the treatment of compressive myelopathies, including multilevel cervical spondylotic myelopathy, ossification of the posterior longitudinal ligament (OPLL), cervical spinal canal stenosis, and degenerative disc herniation (Lau et al., 2017; He et al., 2024). Posterior lateral fusion (PLF) enables neural decompression through laminectomy or laminoplasty while maintaining postoperative stability with internal fixation systems such as lateral mass screw–rod constructs (Wang et al., 2021; Singh et al., 2003; Vedantam et al., 2023). As the use of this technique has expanded, increasing attention has been given to differences among surgical approaches in postoperative spinal stability, neurological recovery, and the risk of adjacent segment degeneration (ASD) (Zhang et al., 2023). These differences may be related to the extent of decompression, fixation stiffness, and intraoperative soft-tissue management. However, the biomechanical basis for these variations remains incompletely understood (Guan et al., 2023; Habibi et al., 2022). In clinical practice, optimizing surgical strategies to balance effective neural decompression with sufficient mechanical stability remains a major challenge in cervical spine surgery.

Traditional biomechanical investigations of the spine have largely relied on cadaveric experiments, which have provided important insights into the mechanical principles of cervical surgical procedures (Ansaripour et al., 2022). However, their application is limited by several inherent drawbacks. Anatomical variability among specimens reduces reproducibility and makes it difficult to systematically examine the relationship between surgical technique and mechanical behavior (Singh et al., 2003). In addition, most cadaveric studies are performed under static loading conditions and cannot adequately reflect the dynamic mechanical behavior or long-term biomechanical evolution of the cervical spine during physiological motion (Yoganandan et al., 1990). The limited preservation time and usability of cadaveric specimens further restrict simulation of progressive postoperative degeneration (Puttlitz et al., 2004). These limitations highlight the need for advanced digital biomechanical modeling approaches with high precision, strong reproducibility, and greater flexibility than traditional experimental methods.

Finite element analysis (FEA) has emerged as a core tool in spinal biomechanics because of its high modeling accuracy and capacity to simulate complex loading conditions (Ahmadi et al., 2025). By integrating high-resolution magnetic resonance imaging (MRI) and computed tomography (CT) data, finite element (FE) techniques enable the construction of individualized cervical spine models incorporating vertebrae, intervertebral discs, and ligamentous structures, thereby reproducing patient-specific anatomy and biomechanical responses with high fidelity (Nikkhoo et al., 2019; Herron et al., 2020). Recent studies have applied FEA to evaluate intradiscal pressure, postoperative stability after laminar decompression, and the mechanical performance of fixation systems (Zafarparandah et al., 2013). However, existing studies have not sufficiently considered the differences between intraoperative decompression techniques, such as the effects of laminoplasty and laminectomy on stress distribution in soft and hard tissues or on ASD (Harinathan et al., 2024). In addition, most studies have not adequately incorporated dynamic loading, muscular forces, or progressive degeneration (Li et al., 2024), which limits understanding of the long-term biomechanical consequences and potential risks of these surgical procedures.

Laminectomy and laminoplasty are two widely used posterior cervical decompression techniques that differ in the extent of anatomical disruption, degree of neural decompression, and preservation of posterior elements. Laminectomy enlarges the spinal canal by removing the lamina, but this approach may substantially impair spinal stability (Kode et al., 2014), increase the mechanical burden on fixation devices, and accelerate biomechanical imbalance in adjacent segments (Vedantam et al., 2023). In contrast, laminoplasty preserves part of the ligamentous complex, which may maintain cervical stability, although its decompression effect can be relatively limited (Tadepalli et al., 2011). However, postoperative changes in multidirectional motion, stress concentration, and fatigue-related load transfer in adjacent segments remain poorly understood (Guan et al., 2023; Habibi et al., 2022). Although preliminary hypotheses and case reports provide limited support for these assumptions, systematic FE analyses and quantitative studies are still lacking, which hinders evidence-based optimization of surgical strategies and clinical decision-making (Liu G. et al., 2024).

ASD is a common complication following posterior cervical surgery, and its pathogenesis involves multiple factors, including altered stiffness of the fused segments, stress-shielding effects, and abnormal redistribution of range of motion (ROM) (Harinathan et al., 2024). Although several studies have used FE modeling to investigate the mechanisms of ASD (Yu Q. et al., 2024), many existing models remain overly simplified, often failing to account for the complex contributions of soft tissues and the dynamic mechanical changes of the postoperative cervical spine (Jiang and Li, 2019). In addition, individualized FE models based on high-resolution imaging that accurately characterize the biomechanical effects of different decompression extents and fixation strategies on ASD remain limited (Liu Y.-T. et al., 2024). Integrating predictive mechanical parameters, such as peak intradiscal pressure and ligamentum flavum (LF) tensile stress, with postoperative MRI findings to identify early quantitative markers and dynamic patterns of degeneration may represent an important direction for future research. These coupled analyses could provide a more comprehensive understanding of the mechanical consequences of different surgical techniques.

The present study aimed to construct an individualized three-dimensional FE model of the cervical spine by integrating high-resolution MRI and CT data while incorporating soft-tissue loading and muscular mechanical effects. Using this model, biomechanical stability, intervertebral disc stress distribution, and the risk of ASD were systematically compared between laminoplasty and laminectomy. In addition, biomechanical simulation results were combined with postoperative imaging data to characterize the long-term mechanical consequences of the two surgical approaches and to establish a degeneration risk prediction framework based on key mechanical parameters. Multiple biomechanical indicators, including intradiscal pressure (IDP), facet joint contact stress, and LF tensile stress, were analyzed to identify a potential balance between decompression efficacy and mechanical stability. These findings may provide quantitative evidence to support surgical decision-making and offer a novel framework for individualized, precision-based strategies in posterior cervical surgery, with the goal of improving postoperative outcomes and long-term quality of life.

In this study, the analytical modules were hierarchically structured to test a unified biomechanical hypothesis. We hypothesized that the choice of posterior decompression procedure would first determine the extent of posterior structural preservation, thereby influencing global and segmental ROM, redistributing local stresses in the intervertebral disc, facet joint, LF, and fixation system, and ultimately affecting adjacent-segment degeneration and its MRI-detectable manifestations. Based on this mechanistic chain, degeneration-related biomechanical indicators were further integrated into a postoperative risk prediction framework. This design was intended to progress from structural alteration to mechanical consequences, clinical validation, and finally translational prediction, rather than applying multiple analytical techniques.

## Materials and methods

### Ethical statement

This study employed a research design combining retrospective imaging data collection with prospective numerical simulation analysis, ensuring that model construction was grounded in authentic clinical imaging data and exhibited high reproducibility across multiple biomechanical parameters. The study was conducted in accordance with the Declaration of Helsinki and approved by the institutional ethics committee (No.: 2025-(8)). Written informed consent was obtained from all participants before imaging acquisition, and additional consent was obtained from patients who underwent postoperative imaging follow-up for the use of their data in subsequent analyses. The study involved only human imaging data and numerical simulations and included no animal experiments or clinical interventions.

To ensure methodological rigor, predefined inclusion and exclusion criteria were applied during participant selection to identify subjects with anatomically normal cervical structures and adequate imaging quality. Standardized imaging protocols were implemented to control scanning resolution and minimize motion or reconstruction artifacts. During the numerical simulation stage, all surgical models and loading conditions were predetermined before analysis to reduce subjective bias. In the statistical analysis stage, multifactorial modeling and validation approaches were applied to evaluate the relationships between biomechanical parameters and imaging outcomes, thereby enhancing the robustness and reliability of the analytical results.

### Participant selection

Study participants were selected based on rigorous anatomical and imaging criteria. All imaging data were retrieved from the institutional clinical imaging database between January 2022 and December 2023. The inclusion criteria were as follows: age between 20 and 40 years; no history of cervical trauma or surgery; no congenital spinal deformity or systemic bone disease; and no apparent degenerative changes on imaging in subjects included for FE modeling. These datasets were used to reconstruct anatomically representative cervical spine models for biomechanical simulation. A separate clinical cohort of postoperative patients was used for imaging analysis to evaluate potential degenerative changes in adjacent segments after surgical treatment. All imaging datasets were required to meet strict quality standards, including high spatial resolution (CT slice thickness ≤0.5 mm; MRI T2 slice thickness ≤2 mm) and minimal artifacts, to ensure accurate geometric reconstruction for subsequent modeling. Exclusion criteria included poor image quality, intervertebral space narrowing, significant LF hypertrophy, osteophyte formation, or any other condition that might affect cervical spine biomechanics. All imaging screening and interpretation were independently performed by two radiologists with more than 10 years of experience and then cross-validated by a third senior expert to ensure consistency and reproducibility.

A total of 30 participants were ultimately included (mean age 31.2 ± 5.3 years). Participants were divided into three independent groups according to the surgical simulation scenario: the intact-model group, the laminectomy-model group, and the laminoplasty-model group (n = 10 per group). Each group consisted of different individuals, and no longitudinal pre–post comparison was performed. Instead, the grouping represented distinct simulated surgical configurations rather than temporal changes within the same subjects. High-resolution FE models of the C3–C7 segments were constructed for all participants, while the adjacent segments C2–C3 and C7–T1 were included to evaluate stress transmission and potential degeneration trends. The three groups demonstrated comparable distributions in sex ratio, age range, and imaging quality. Imaging quality was assessed by experienced radiologists based on resolution consistency, absence of severe artifacts, and clear anatomical boundaries suitable for precise geometric reconstruction, thereby minimizing modeling bias and ensuring the reliability of biomechanical simulations.

### Imaging data acquisition

Imaging was acquired using a standardized protocol. CT scans were performed on a SOMATOM Definition Flash system (Siemens Healthineers) or equivalent equipment, with parameters set at 120 kVp, automatic mAs modulation, and a voxel size of ≤0.5 × 0.5 × 0.5 mm. MRI was performed on a MAGNETOM Skyra 3.0T scanner using T2-weighted sagittal and axial sequences, with repetition time/echo time (TR/TE) values defined according to the clinical standard protocol, slice thickness ≤2 mm, and unified field-of-view and matrix settings. Postoperative follow-up imaging was obtained at 0, 3, and 6 months to capture the early dynamic features of postoperative degeneration. All imaging examinations were conducted using fixed acquisition protocols by the same technical team to minimize systematic variability and temporal bias. After acquisition, all DICOM datasets were automatically de-identified and converted to NIfTI format for subsequent registration, model construction, and statistical analysis (Supplementary Figure S1).

### Geometric reconstruction and mesh generation

Geometric reconstruction of the cervical spine FE model was performed using high-resolution CT data according to a standardized image-processing workflow to ensure accurate anatomical representation. During segmentation and surface reconstruction, osseous structures were delineated in Mimics (v24.0, Materialise) using threshold-based segmentation combined with region growing, boundary tracing, and manual refinement. This procedure enabled extraction of key anatomical components, including vertebral bodies, articular processes, laminae, spinous processes, intervertebral discs, cartilaginous endplates, and facet cartilage. The resulting three-dimensional surface models were then imported into 3-matic (v16.0) for geometric optimization, including surface smoothing, noise reduction, hole filling, and correction of boundary discontinuities, thereby removing local artifacts and irregular edges introduced during image segmentation. Ligament attachment sites, including those of the anterior longitudinal ligament (ALL), LF, and capsular ligament (CL), were identified on the basis of imaging features and established anatomical landmarks. These sites were independently annotated by two biomechanical modeling researchers and verified by a third expert to ensure anatomical accuracy and structural consistency.

During voxel-to-solid conversion and mesh generation, tissue-specific meshing strategies were applied to balance computational accuracy and efficiency. Mesh generation was performed in ANSYS Workbench, and the assembled models were subsequently imported into ANSYS Mechanical for verification and numerical analysis. Cancellous bone was primarily discretized using second-order tetrahedral solid elements, which are well-suited to the curved surfaces and irregular geometry of vertebral and posterior osseous structures. Cortical bone was modeled using thin-layer solid elements or equivalent shell elements (e.g., SHELL181 or thin-layer implementations of SOLID185/SOLID186), preserving structural stiffness while reducing the overall element count and improving computational stability. For the intervertebral disc, a hexahedral-dominant mesh was employed to reduce volumetric locking and enhance numerical stability in stress and pressure calculations, consistent with previously validated cervical spine FE studies showing that hybrid tetrahedral–hexahedral meshing provides a reasonable balance between geometric fidelity and numerical robustness (Nikkhoo et al., 2019; Herron et al., 2020; Zafarparandah et al., 2013). The annulus fibrosus was modeled as a multilayer concentric structure with collagen fibers arranged in alternating orientations of approximately ±30° relative to the transverse plane, consistent with anatomical alignment. Its anisotropic mechanical behavior was represented using fiber-reinforced formulations or direction-dependent material parameters. This approach was adopted to better reproduce the anisotropic load-bearing behavior of the annulus fibrosus and was based on the established fiber architecture commonly used in validated spinal FE models (Nikkhoo et al., 2019; Herron et al., 2020). Facet joint cartilage and contact regions were locally refined, with a target element size of 0.3–0.5 mm to ensure accurate calculation of contact pressures and stress concentrations, particularly in segments such as C5–C6. Such local refinement is commonly used in spinal FE analysis to improve the reliability of contact-pressure estimation in mechanically sensitive regions (Zafarparandah et al., 2013; Nikkhoo et al., 2019). Prior to assembly of the integrated model, all components underwent comprehensive geometric and mesh quality control, including connectivity verification, correction of free edges and non-manifold geometries, elimination of element overlap or penetration, and enforcement of threshold criteria for element quality metrics such as skewness, aspect ratio, and Jacobian determinants. These procedures minimized numerical instability and prevented non-convergence or artificial stress concentrations caused by mesh distortion or geometric discontinuities.

### Mesh density and convergence verification

To ensure the numerical stability and reproducibility of the FE model, mesh statistics were evaluated after mesh generation. The final assembled finite element model of the cervical spine consisted of approximately 620,000 elements and 910,000 nodes, providing sufficient resolution to capture its complex anatomical geometry and stress distribution. Vertebral bodies and posterior osseous structures were primarily discretized using second-order tetrahedral elements, whereas the intervertebral discs were meshed predominantly with structured hexahedral elements to improve numerical accuracy in pressure and deformation calculations. The material models and corresponding parameters used in the FE model are summarized in Supplementary Table S1. Supplementary Table S1 provides the specific numerical parameter values, constitutive formulations, contact/interface definitions where applicable, and the corresponding supporting references for each modeled component.

A mesh convergence analysis was performed to verify that computational results were independent of mesh density. Three mesh densities (coarse, medium, and fine) were generated through systematic refinement of element size. The maximum IDP at the C5–C6 segment under flexion loading was selected as the primary biomechanical indicator for convergence evaluation, because this segment is commonly subjected to relatively high mechanical demand in cervical FE analyses and has been widely used as a representative parameter for evaluating disc loading sensitivity in previous studies (Zafarparandah et al., 2013; Nikkhoo et al., 2019). The difference in maximum IDP between the medium- and fine-density meshes was less than 5%, indicating that the medium-density mesh achieved sufficient accuracy while preserving computational efficiency. Accordingly, the medium-density mesh was adopted for all subsequent simulations.

A hybrid meshing strategy was intentionally adopted. Due to the highly irregular geometry of vertebral structures, tetrahedral elements were used to accurately represent complex anatomical morphology. In contrast, the intervertebral discs were modeled with hexahedral-dominant meshes to improve numerical stability for nearly incompressible materials and to reduce volumetric locking during pressure calculations. Previous spinal FE studies have demonstrated that such hybrid meshing approaches effectively balance geometric fidelity, computational efficiency, and numerical robustness.

To minimize potential numerical errors associated with mixed-element meshes, strict mesh quality criteria were applied, including thresholds for skewness, aspect ratio, and Jacobian determinant. Contact regions, such as the facet joints and endplates, were also locally refined to improve the accuracy of stress estimation. These procedures ensured stable numerical convergence and reliable stress distribution throughout the simulations.

### Validation of the normal model

The reconstructed three-dimensional FE model of the normal cervical spine (C2–C7) was imported into ANSYS Workbench, where boundary conditions and loading protocols were defined. All degrees of freedom at the inferior endplate of C7 were constrained to replicate the lower-end fixation commonly used in *in vitro* biomechanical experiments.

Pure moment loading, which is widely used in cervical spine biomechanical studies, was applied to the superior end to simulate physiological cervical motion. For model validation, loading conditions included flexion/extension (±1.0 N m), lateral bending (±1.0 N m), and axial rotation (±1.0 N m), because pure moments of this magnitude have been widely adopted in cervical *in vitro* and FE validation studies of the cervical spine, allowing direct comparison with previously reported segmental ROM data under physiological motion conditions (Jayakumar, 2018; Xing et al., 2020; Xie et al., 2021). When physiological compressive preload was considered, a 75 N follower load was applied along the curvature of the cervical spine to represent the combined compressive effects of head weight and muscle forces. To reduce numerical instability associated with nonlinear material behavior and contact interactions, loads were applied incrementally using a stepwise quasi-static loading scheme (Supplementary Figure S2). Similar loading protocols have been widely adopted in validated cervical spine FE studies.

The primary biomechanical outputs were segmental ROM and IDP for each intervertebral disc. ROM was calculated from the angular displacement of individual vertebrae under the applied loads. IDP was derived from the stress distribution within the nucleus pulposus. Specifically, the average hydrostatic pressure of all nucleus pulposus elements was extracted from the ANSYS output corresponding to compressive pressure, and the mean value was used to represent the IDP of each disc. This method has been widely used in previous cervical spine FE studies to assess disc-loading conditions.

To verify model reliability, the simulated ROM and pressure values were compared with cadaveric experimental reference data reported by White and Panjabi (1990) (Evans, 1992). Model accuracy was evaluated using relative error, with ±10% defined as the acceptable threshold, consistent with validation criteria commonly adopted in FE biomechanical studies.

To further confirm biomechanical plausibility, stress distributions in osseous, articular, and ligamentous structures were continuously monitored throughout the simulations. Patterns of intervertebral disc pressure were examined to ensure consistency with known cervical biomechanical responses under different motion conditions and to exclude unrealistic stress concentration at disc margins. When abnormal results were identified, iterative adjustments were performed, with priority given to refinement of geometric reconstruction, joint-gap and contact definitions, material parameters, and mesh quality, until the validation criteria were met.

### Surgical modeling

To simulate the morphological changes induced by laminoplasty and laminectomy, a three-dimensional FE model incorporating the normal laminar structure was constructed. Geometric structures were first extracted and reconstructed from medical imaging data using Mimics, followed by surface refinement and mesh optimization in Geomagic. In the laminectomy model, the posterior laminae at the decompressed segments were completely removed to represent full posterior decompression, and the corresponding LF attachments were deleted. In contrast, the laminoplasty model preserved the laminar structure and introduced a simulated hinge-opening configuration, thereby enlarging the spinal canal while maintaining partial integrity of the posterior elements. These geometric modifications were defined according to commonly used surgical procedures and previously reported experimental and computational studies. These procedure-specific geometric definitions were adopted to preserve the key structural differences between laminectomy and laminoplasty, particularly the extent of posterior element removal versus preservation, which directly influences postoperative stability and adjacent-segment loading (Kode et al., 2014; Tadepalli et al., 2011; Zafarparandah et al., 2013).

Internal fixation devices were incorporated according to the Magerl lateral mass screw technique. Screw diameter was set at 3.5 mm, and screw length ranged from 14 to 16 mm, consistent with reported anatomical and surgical parameters (Singh et al., 2003; Vedantam et al., 2023). In the ANSYS FEA environment, interactions between metallic fixation components and bone tissues were defined using surface-to-surface contact formulations with an assigned friction coefficient to simulate screw–bone interface behavior (Ansaripour et al., 2022; Wang et al., 2021). Bone tissues were assigned anisotropic material properties, whereas the fixation devices were modeled as isotropic titanium alloy (Nikkhoo et al., 2019; Herron et al., 2020). Ligamentous structures and intervertebral discs were assigned material parameters within ranges reported in previously validated cervical spine FE models to ensure biomechanical consistency with experimental observations (Yu Q. et al., 2024; Jiang and Li, 2019). Comparative simulations were performed for short-segment fixation (C3-C5) and long-segment fixation (C2-C7) under identical axial loading and torsional conditions to assess the influence of fixation range on segmental stability and stress distribution.

To improve the biological relevance of the simulations, postoperative clinical imaging data from laminoplasty and laminectomy cases were used as references. Structural stability was assessed using parameters including laminar thickness, screw-loosening tendency, and flexion–extension behavior of the fixation segments. Integration of biomechanical simulation results with clinical imaging observations enabled further evaluation of the advantages and potential risks associated with each surgical approach and provided a theoretical basis for predicting postoperative biomechanical outcomes.

### Simulation loading and boundary condition settings

In the cervical spine biomechanical simulations, loading conditions were defined in accordance with ASME international guidelines to reproduce physiological cervical motion. To simulate typical motion modes, including flexion, extension, lateral bending, and axial rotation, six-degree-of-freedom physiological loads were applied to the superior surface of the C3 vertebra. A 75 N follower compressive load was first imposed to represent the combined effects of head weight and baseline muscular tension. This follower-load strategy was adopted as a practical approximation of physiological compressive loading because it allows the cervical spine to sustain preload while minimizing artificial bending moments during motion simulation (Jayakumar, 2018; Xing et al., 2020; Xie et al., 2021). Pure moments of ±1.0 N m were subsequently superimposed to generate flexion, extension, lateral bending, and axial rotation.

For the postoperative models, the stabilizing contribution of the posterior cervical musculature was represented indirectly through follower loading and preservation of the ligamentous system rather than through simplified active muscle elements. This approach improves computational stability while retaining the major passive stabilizing mechanisms commonly adopted in cervical FE studies (Yu Q. et al., 2024; Jiang and Li, 2019). The modeled ligaments included the ALL, PLL, LF, interspinous ligament, and CLs. These structures were assigned nonlinear tension-only material properties based on stress–strain relationships reported in previously validated cervical spine FE models. To enhance numerical stability and avoid convergence problems under different motion conditions, all loads were applied incrementally using a stepwise loading scheme.

For boundary conditions, all degrees of freedom at the inferior surface of the C7 vertebra were fully constrained. To prevent nonphysiological penetration or artificial fusion of the facet joints, the articular surfaces were defined using surface-to-surface contact formulations. Normal contact behavior was governed by a penalty-based contact algorithm, and a tangential friction coefficient of 0.1 was assigned to permit realistic sliding and separation between joint surfaces. The intervertebral disc was modeled as a composite structure. The nucleus pulposus was represented as a nearly incompressible hyperelastic material, whereas the annulus fibrosus matrix was assigned hyperelastic properties and reinforced with two families of tension-only collagen fibers to capture anisotropic behavior. Fiber orientations were defined at approximately ±30° relative to the transverse plane, consistent with the anatomical arrangement of annular fibers.

### Global and local stability analysis

A refined FEA model of the C2–C7 cervical spine segments was constructed to evaluate the effects of different decompression ranges on spinal stability. The model was reconstructed from clinical imaging data to accurately reproduce the geometric features of both osseous and soft-tissue structures. Nonlinear material models were assigned to soft tissues, including ligamentous structures, to better reflect their physiological mechanical behavior. Surgical decompression was simulated by removing designated posterior osseous regions and modifying the corresponding spinal canal contact relationships to reproduce clinically relevant anatomical changes. Physiological loading conditions, including flexion, extension, lateral bending, and axial rotation, were then applied. Postoperative ROM at each cervical segment was calculated to quantitatively assess the effects of decompression range on both global and segmental stability.

To evaluate fixation performance, a refined FE analysis of the screw–bone interface was performed. Variations in axial stress, shear force, and interfacial contact pressure were analyzed under different decompression scenarios. Mechanical responses at each screw position were extracted, and changes in screw–bone contact area were incorporated to assess fixation stability. Bone density at screw insertion sites was defined according to the local Hounsfield unit distribution, allowing simulation of screw mechanics under different bone quality conditions. In addition, decompressed and non-decompressed segments were compared to examine load transmission patterns and their influence on overall cervical stability. This approach enabled evaluation of how surgical decompression alters load redistribution across the cervical spine.

All mechanical outputs were first compared with previously published experimental data to confirm biomechanical plausibility. Multivariate analyses incorporating decompression range and screw position were then conducted under specific surgical scenarios to explore strategies for optimizing cervical stability while maintaining effective neural decompression. By integrating the mechanical responses of osseous and soft-tissue structures under physiological loading, the model provides a biomechanical framework for determining appropriate decompression ranges and fixation parameters.

The biomechanical indicators evaluated in this study were selected on the basis of commonly used metrics in cervical spine FE research. These included ROM, IDP, facet joint contact stress, LF tension, and screw–bone interface stress. ROM was used to characterize global and segmental stability, whereas IDP served as an indicator of disc loading and degeneration risk. Facet joint contact stress reflects load redistribution within posterior spinal elements, LF tension represents the mechanical response of posterior ligamentous structures during motion, and screw–bone interface stress was used to evaluate fixation stability and the potential fatigue risk of the implant system. These parameters have been widely adopted as standard biomechanical indices in validated cervical spine FE studies (Zafarparandah et al., 2013; Nikkhoo et al., 2019; Herron et al., 2020).

### Simulation of ASD

To simulate the biomechanical behavior of the superior and inferior adjacent segments under degenerative conditions, intervertebral disc degeneration parameters were adjusted according to the Panjabi degeneration model and previously validated FE studies, as this framework captures the characteristic transition from nucleus softening to annular stiffening during progressive degeneration (Kiliçkan et al., 2005; Pind, 1998). Specifically, the elastic modulus of the nucleus pulposus in the adjacent segments was reduced to 20% of its original value to represent the loss of load-bearing capacity and material softening caused by dehydration. In contrast, the elastic modulus of the annulus fibrosus was increased to 150% of its initial value to simulate the stiffening associated with the fibrotic stabilization phase of degeneration. These parameter adjustments were intended to reproduce the reduced hydrostatic support of the dehydrated nucleus pulposus and the compensatory fibrotic stiffening of the annulus fibrosus described in degeneration-oriented FE studies (Kiliçkan et al., 2005; Pind, 1998). The intervertebral disc was modeled using an incompressible Neo-Hookean hyperelastic formulation. Although more complex constitutive models have been proposed, this formulation was adopted to maintain numerical stability and computational efficiency in the multilevel instrumented cervical model. Previous studies have shown that the Neo-Hookean model provides a reasonable approximation of the hydrostatic response of the nucleus pulposus and the nonlinear mechanical behavior of the annulus fibrosus within physiological strain ranges (Nikkhoo et al., 2019; Herron et al., 2020). These parameter adjustments were designed to represent the progressive biomechanical changes associated with disc degeneration before and after surgical intervention.

During FE model construction, the adjacent segments C2–C3 and C7–T1 were reconstructed from CT imaging data, and degeneration-related parameters were incorporated to enable dynamic assessment of IDP and endplate stress distribution. Physiological moments and shear loads were applied incrementally to simulate both normal motion and postoperative biomechanical responses under different movement patterns. The loading protocol included flexion, extension, lateral bending, and axial rotation, together with axial compression, allowing evaluation of stress variation and load redistribution under complex physiological conditions.

Stress distribution maps were further analyzed to evaluate pressure gradients within the C2–C3 and C7–T1 intervertebral discs, as well as localized endplate stress amplification, in order to identify potential biomechanical factors contributing to postoperative adjacent segment degeneration (Supplementary Figure S3).

### Clinical imaging analysis and validation

To further evaluate the clinical relevance of the biomechanical findings, a separate retrospective cohort of postoperative patients who underwent cervical laminoplasty or laminectomy was analyzed using MRI. This clinical imaging cohort was independent of the FE modeling cohort and was used to assess postoperative degenerative changes in adjacent intervertebral discs. Disc degeneration was graded according to the Pfirrmann classification system to provide a standardized quantitative assessment of degeneration severity. All MRI examinations were performed using standardized acquisition protocols, with high-resolution sequences applied to evaluate intervertebral discs and adjacent soft tissues, including the LF, thereby ensuring image consistency and comparability. Each imaging dataset was independently assessed by two experienced radiologists, and interobserver reliability was confirmed by consistency analysis. During LF annotation, both thickness and T2-weighted signal intensity changes were recorded as reference indicators of degeneration.

The imaging datasets were systematically organized and processed using customized analysis scripts to extract key parameters related to adjacent segment degeneration. These parameters included degeneration area ratios and imaging features associated with stress-related structural alterations. In parallel, the FE model was used to estimate stress distributions within the intervertebral discs and surrounding soft tissues. The clinical FE model incorporated postoperative anatomical variations, including changes in intervertebral height, marginal osseous remodeling, and alterations in soft-tissue integrity. Comparative analyses were then performed to examine the spatial correspondence between regions of elevated stress predicted by the FE model and degenerative features identified on MRI. Multivariate trend analyses further explored the associations among imaging-derived parameters, mechanical stress distributions, and degeneration severity.

To further validate the predicted high-stress regions, LF thickening and reduced T2-weighted signal intensity were used as additional calibration indicators. The spatial distribution of these features across the dataset was analyzed to characterize LF degeneration patterns. Regional correlation matrix analyses were subsequently performed to examine relationships across different degeneration grades. This integrated imaging–biomechanical approach enabled evaluation of the predictive performance of the FE model and clarified the clinical significance of MRI-based structural annotations in assessing postoperative degeneration and disease progression.

### Fatigue simulation analysis of the fixation system

A cyclic loading simulation was performed to evaluate the fatigue behavior of the fixation system during long-term use. The loading frequency was set at 1–2 Hz, corresponding to the typical frequency of cervical motion during daily activities and biomechanical experiments (Hamada et al., 2018; Fuentes et al., 2001). The loading amplitude was maintained within the physiological range, varying periodically between 100 N and 300 N, and the simulation was performed for more than 10^5^ loading cycles. Fracture mechanics–based models were subsequently applied to estimate fatigue-related crack propagation and relative fixation stability under cyclic loading. The analysis focused on equivalent stress distribution, crack propagation behavior, hysteresis response, and residual displacement at the screw–bone interface, thereby enabling comparative evaluation of fatigue-related mechanical stability between the laminoplasty and laminectomy models.

### Statistical analysis

To minimize the influence of inter-individual anatomical variability on biomechanical outcomes, several methodological controls were implemented. Strict inclusion criteria were applied to ensure relatively homogeneous cervical anatomy among participants, including the absence of spinal deformities, trauma, or significant degenerative changes on imaging. All FE models were reconstructed using standardized imaging protocols, segmentation procedures, and meshing workflows to ensure structural consistency. In addition, identical boundary conditions and loading protocols were applied in all simulations, including a 75 N follower load combined with pure moments of ±1.0 N m, thereby allowing direct comparison of biomechanical responses among surgical scenarios. Continuous biomechanical parameters were normalized before statistical analysis to reduce the effect of inter-subject variability.

All data were analyzed using Prism 10.0 software. Continuous variables are presented as mean ± standard deviation (SD). Because the datasets represented independent FE models derived from different individuals, comparisons among the three groups were performed using one-way analysis of variance (ANOVA). When significant differences were detected, Bonferroni *post hoc* tests were used for pairwise comparisons. For comparisons between two groups, independent-sample t-tests were applied. A *p*-value <0.05 was considered statistically significant. The relationship between FE-predicted parameters and clinical imaging findings was assessed using Spearman rank correlation analysis. To eliminate the influence of different variable scales, all continuous variables were normalized before correlation analysis. Sensitivity and subgroup analyses were further conducted to evaluate the robustness and stability of the model. To enhance analytical reliability, all analyses were independently reviewed, and repeated testing together with multivariate regression modeling was performed to assess potential interactions among key variables and possible confounding effects. For intersegmental comparisons within the Normal Cervical Spine FE Model, repeated-measures one-way ANOVA followed by Bonferroni-adjusted pairwise comparisons was used, because measurements from different cervical segments within the same FE model were not statistically independent; therefore, no within-group t-test was applied for that analysis.

## Results

### Construction and validation of a normal cervical spine FE model demonstrating high consistency in kinematic and mechanical responses

By integrating high-resolution CT and MRI data, a subject-specific three-dimensional FE model of the normal cervical spine was constructed for the C3–C7 segments. The model preserved key anatomical features, including flattened vertebral bodies, obliquely oriented facet joints, and transverse foramina. A hybrid tetrahedral–hexahedral meshing strategy was used to discretize the vertebral bodies, articular surfaces, intervertebral discs (annulus fibrosus and nucleus pulposus), and major ligamentous structures.

**FIGURE 1:**
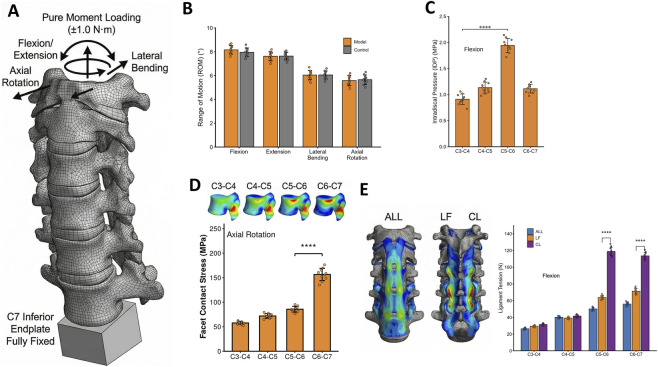
Structural composition and validation analysis of the normal cervical spine FE model. Note: **(A)** A three-dimensional FE model of the normal C3-C7 cervical spine constructed from high-resolution CT and MRI data, illustrating cervical-specific anatomical morphology, mesh discretization, and boundary/loading conditions. Key cervical anatomical features, including flattened vertebral bodies, obliquely oriented facet joints, and transverse foramina, were preserved; the inferior endplate of the C7 vertebra was fully constrained, and pure moments were applied at the superior end to simulate physiological motion. **(B)** Validation of the model by comparing predicted global ROM under flexion, extension, lateral bending, and axial rotation with *in vitro* experimental data reported by White and Panjabi (Control). **(C)** Segment-specific distributions of IDP within the nucleus pulposus. **(D)** Three-dimensional stress contour maps and quantitative analyses of facet joint contact stress across segments under axial rotation. **(E)** Stress contour maps and segment-wise quantitative comparisons of tension in the ALL, LF, and CL under flexion. Data are presented as mean ± SD, N = 10; **p < 0.01, ***p < 0.001, ****p < 0.0001.

For model validation, physiological boundary conditions were applied by fully constraining the inferior endplate of C7 to represent distal support, while standardized pure moments were imposed at the superior end to generate cervical motion. The applied moments were ±1.0 N m for flexion/extension, ±1.0 N m for lateral bending, and ±1.0 N m for axial rotation (Figure 1A).

Under four representative physiological motion modes (flexion, extension, lateral bending, and axial rotation), the overall ROM of the C3-C7 segments was 8.1° ± 0.3°, 7.8° ± 0.4°, 6.3° ± 0.2°, and 5.9° ± 0.3°, respectively. These values closely matched the *in vitro* experimental data reported by White and Panjabi, with relative errors consistently maintained within 8% (Figure 1B), indicating that the model reliably reproduced the global kinematic behavior of the cervical spine.

Further analysis of internal mechanical responses demonstrated physiologically consistent loading patterns. During flexion, IDP within the nucleus pulposus increased progressively across segments, with the highest value observed at C5–C6 (Figure 1C), suggesting that this level bears greater disc loading during sagittal motion. Under axial rotation, facet joint contact stress showed a distinct segmental distribution, with the C6–C7 segment exhibiting markedly higher articular contact stress than the other levels (Figure 1D), suggesting a potential region of stress concentration during rotational motion.

Ligament tension analysis showed trends closely consistent with the distribution of facet joint stress. During flexion, tension in the ALL and LF increased synchronously, whereas peak CL tension was mainly concentrated at C5–C6 (Figure 1E). This segment-dependent mechanical pattern suggests coordinated load sharing between ligamentous structures and facet joints during cervical motion.

Overall, the FE model showed strong agreement with previously reported experimental data in both kinematic behavior and tissue-level mechanical responses. These findings support its reliability as a platform for simulating cervical spine biomechanics and for subsequent analyses of surgical interventions and ASD.

### Laminectomy significantly increases cervical ROM while laminoplasty preserves stability

**FIGURE 2:**
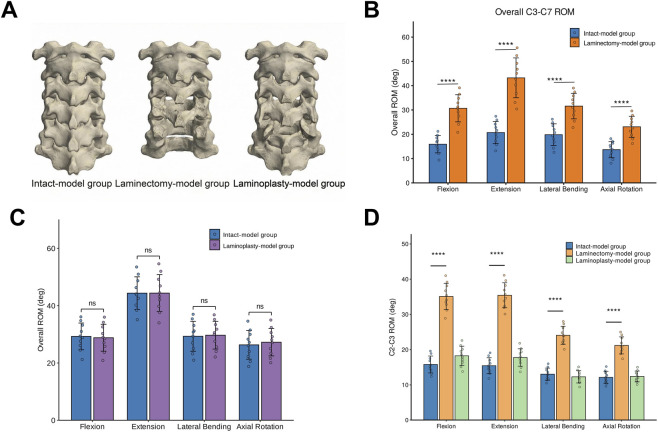
Effects of laminectomy and laminoplasty on postoperative cervical spine ROM. Note: **(A)** Three-dimensional morphological comparison of the intact C3–C7 cervical FE model and the corresponding laminectomy and laminoplasty models constructed from the same baseline geometry. All surgical models were derived directly from the same validated cervical spine geometry, with modifications confined to the posterior laminae and related osseous structures to ensure geometric consistency between procedures. **(B)** Comparison of global cervical ROM between the intact model and the laminectomy model under uniform mechanical loading conditions (flexion/extension ±1.0 N m, lateral bending ±1.0 N m, axial rotation ±1.0 N m). **(C)** Comparison of global cervical ROM between the intact model and the laminoplasty model under the same loading conditions. **(D)** Changes in ROM of the adjacent C2-C3 segment in the intact, laminectomy, and laminoplasty models under identical loading conditions. Data are presented as mean ± SD, N = 10; * indicates comparison with the intact model, *p <* 0.05; **** indicates *p <* 0.0001.

ROM was analyzed first because it is the most direct biomechanical indicator of postoperative global and segmental stability and an upstream determinant of load redistribution after posterior structural alteration. Based on the validated FE model of the normal cervical spine, numerical simulations of different posterior decompression procedures were performed on the same intact C3-C7 cervical geometry to generate postoperative laminectomy and laminoplasty models (Figure 2A). All postoperative models were derived from the same validated geometric framework, with surgical modifications limited to the posterior laminae and related osseous structures, thereby ensuring geometric consistency and comparability between procedures. Under standardized physiological loading conditions, including flexion/extension (±1.0 N m), lateral bending (±1.0 N m), and axial rotation (±1.0 N m), the effects of the two surgical techniques on overall cervical ROM were systematically evaluated. In Figure 2A and the related text, the three simulated configurations are referred to as the intact-model, laminectomy-model, and laminoplasty-model groups to avoid confusion with a longitudinal pre-post patient design.

Compared with the intact model, the overall cervical ROM increased after laminectomy across all motion modes. The greatest increase occurred during extension (Figure 2B), indicating reduced posterior structural restraint following laminar removal. In contrast, the laminoplasty model showed ROM values comparable to the intact condition, with only minimal changes across all motion directions and no evidence of excessive mobility (Figure 2C).

Segmental analysis further revealed distinct kinematic responses in adjacent segments. After laminectomy, ROM at the superior adjacent segment (C2–C3) increased markedly, indicating compensatory motion at the adjacent level. In contrast, no significant change in C2–C3 ROM was observed after laminoplasty (Figure 2D).

Overall, these results indicate that laminectomy increases postoperative cervical mobility at both operated and adjacent levels, whereas laminoplasty more effectively preserves physiological motion and segmental stability.

### Laminectomy induces facet joint stress concentration, while laminoplasty maintains uniform load distribution

**FIGURE 3:**
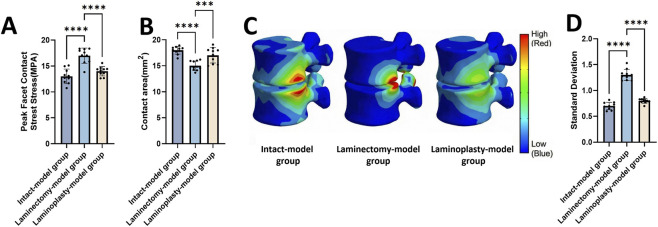
Analysis of the effects of different surgical procedures on facet joint contact stress and contact area. Note: **(A)** FEA of peak facet joint contact stress in the intact-model group, the laminectomy-model group, and the laminoplasty-model group groups; **(B)** FEA results showing facet joint contact area across the same groups; **(C)** Three-dimensional stress distribution maps of C5-C6 facet joints derived from FEA in the intact-model, laminectomy-model, and laminoplasty-model groups, where red indicates regions of high stress concentration and blue denotes low-stress regions; **(D)** SD of peak facet joint contact stress measured by FEA among the three groups. N = 10; ***p < 0.001; ****p < 0.0001 between groups.

Facet joint contact stress was then evaluated to characterize posterior load redistribution after decompression, as abnormal facet loading may reflect mechanical compensation secondary to altered ROM. Under standardized axial rotational loading (10 N m), high-resolution FE simulations were performed to evaluate the effects of laminectomy and laminoplasty on facet joint contact mechanics at the C5–C6 segment. Clear differences were observed between the two procedures in both stress distribution and contact characteristics. Peak facet joint contact stress increased markedly after laminectomy compared with the intact condition, whereas the peak stress after laminoplasty remained comparable to that of the intact-model model (Figure 3A).

Contact area analysis further showed that laminectomy reduced the effective facet joint contact area, accompanied by an increase in peak stress. In contrast, the contact area after laminoplasty remained similar to that of the intact model (Figure 3B).

Three-dimensional stress contour maps demonstrated that, after laminectomy, stress concentration was mainly localized to the medial and posterior–superior margins of the C5–C6 facet joints (Figure 3C). These regions correspond to sites frequently associated with facet degeneration and mechanical irritation in clinical observations. Repeated simulations confirmed the reproducibility of these findings. The laminectomy-model group showed higher mean contact stress and standard deviation than the laminoplasty-model group, while overall variability remained below 1.6 MPa (Figure 3D), indicating good model stability and repeatability.

Overall, these results indicate that laminectomy leads to localized facet joint stress concentration, whereas laminoplasty preserves a more uniform stress distribution across the articular surfaces.

### Laminectomy significantly increases intervertebral disc pressure and induces stress migration, while laminoplasty maintains central equilibrium distribution

IDP was further analyzed because intradiscal loading is closely associated with adjacent-segment mechanical burden and degeneration risk. To evaluate the effects of different surgical procedures on the postoperative disc mechanical environment, an intensified flexion load (10 N m) was applied to the validated cervical FE model. IDP distribution within the nucleus pulposus was examined across the C4–T1 segments, including the cervicothoracic junction. This elevated loading condition was used to amplify intersegmental load transfer and pressure redistribution for mechanistic analysis.

**FIGURE 4:**
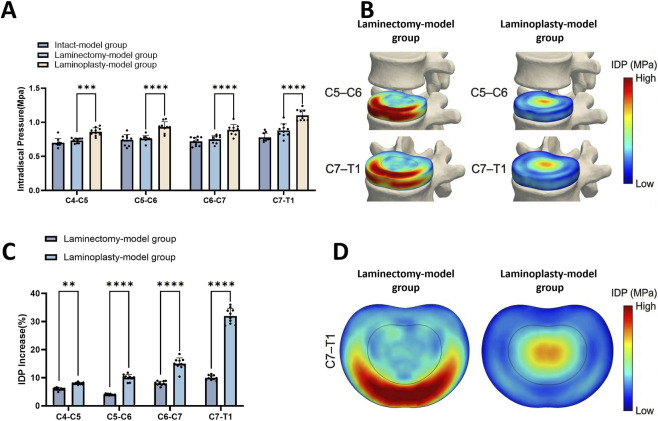
Analysis of the effects of different surgical procedures on IDP distribution. Note: **(A)** Using the same validated cervical FE geometry, peak IDP within the nucleus pulposus was compared across the C4-T1 segments under a 10 N m flexion loading condition for the intact, laminectomy, and laminoplasty models. The cervicothoracic junction was included to evaluate postoperative load transfer effects. **(B)** Three-dimensional contour maps illustrating segment-specific IDP distributions after laminectomy and laminoplasty. **(C)** Percentage increases in IDP across segments for the two surgical procedures, calculated as IDP increase (%) = (post - pre)/pre × 100%. **(D)** Comparison of transverse cross-sectional IDP distribution patterns between the laminectomy and laminoplasty models, with red indicating regions of high pressure concentration and blue indicating low-pressure regions. Data are presented as mean ± SD, N = 10; **p < 0.01, ***p < 0.001, ****p < 0.0001.

Following laminectomy, marked IDP peaks were observed at C5–C6 and C7–T1, reaching 1.27 ± 0.05 MPa and 1.08 ± 0.04 MPa, respectively. These values represented increases of 22.6% and 35.4% relative to the intact model (*p* < 0.01), with distinct high-pressure concentration zones appearing in the distal segments (Figure 4A). In contrast, the laminoplasty model showed substantially smaller pressure changes, with increases of 12.9% at C5–C6 and 3.4% at C7–T1, remaining markedly lower than those in the laminectomy model.

Intersegmental IDP patterns further revealed clear differences in load transfer between the two procedures (Figures 4B,C). After laminectomy, IDP increased progressively from proximal to distal cervical levels, with the highest pressure observed at C7–T1, indicating increased compensatory loading in distal segments. In contrast, the laminoplasty model maintained a relatively uniform pressure gradient across segments, with only minor IDP fluctuations in the C4–C6 region.

Pressure contour analysis also revealed clear differences in the spatial distribution of high-pressure regions (Figure 4D). In the laminectomy model, high-pressure zones shifted from the central nucleus pulposus toward the posterolateral annulus fibrosus and were accompanied by steeper local pressure gradients. In contrast, the laminoplasty model preserved a centrally concentrated pressure pattern within the nucleus pulposus, with an approximately axisymmetric distribution and limited peripheral stress migration.

Overall, these results demonstrate that laminectomy increases IDP and promotes stress redistribution toward distal segments, whereas laminoplasty maintains a more stable intradiscal mechanical environment.

### Laminectomy significantly increases LF tension and stress concentration, while laminoplasty maintains uniform force distribution

**FIGURE 5:**
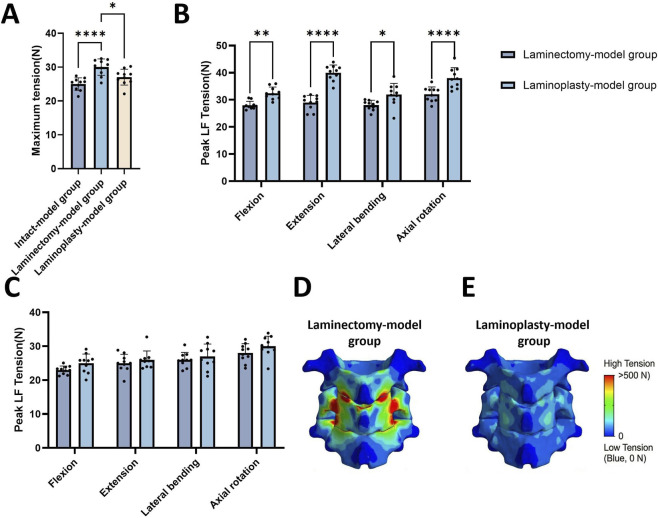
Analysis of LF tensile responses under different surgical models. Note: **(A)** FEA-derived maximum LF tension under static conditions in the intact, laminectomy, and laminoplasty groups. **(B)** Peak LF tension measured by FEA in the intact and laminectomy-model models under flexion, extension, lateral bending, and axial rotation conditions. **(C)** Peak LF tension measured by FEA in the intact-model and laminoplasty-model models under flexion, extension, lateral bending, and axial rotation conditions. **(D,E)** Posterior-view tension contour maps of the LF at the C4-C6 cervical segments, with red indicating regions of high tension concentration and blue indicating low-tension regions. Pronounced high-stress concentration was observed at the C4-C6 levels in the laminectomy-model group **(D)**, whereas the laminoplasty-model group exhibited a more uniform stress distribution without distinct concentration hotspots **(E)**. Data are presented as mean ± SD, N = 10; * indicates between-group comparisons, *p <* 0.05; **p < 0.01; ****p < 0.0001.

LF tension was assessed as a ligament-specific indicator of posterior soft-tissue response and as a potential bridge between FE-predicted stress redistribution and MRI-detectable degenerative change. To evaluate the biomechanical effects of different decompression procedures on the LF, three FE models representing the intact-model, laminectomy, and laminoplasty conditions were constructed and analyzed under multiple motion-loading states. Under static conditions, peak LF tension increased markedly after laminectomy compared with the intact model (Figure 5A). In contrast, the laminoplasty model showed only a modest increase, remaining within the physiological range.

Comparative analysis under flexion, extension, lateral bending, and axial rotation further revealed clear differences between the two procedures. The laminectomy-model group exhibited the highest LF tension during extension and rotation, exceeding the physiological safety threshold. Conversely, the laminoplasty-model group showed only minimal fluctuations across all motion states, indicating greater load adaptability (Figures 5B,C).

Stress contour analysis further illustrated distinct spatial patterns between the two surgical conditions. In the C4–C6 LF regions, the laminectomy model showed pronounced stress concentration zones, characterized by localized high-stress accumulation along the posterior ligamentous structure (Figure 5D). In contrast, the laminoplasty model exhibited a more diffuse stress distribution across the same segments, without obvious focal stress concentration (Figure 5E).

Taken together, these findings indicate that laminectomy increases LF tension and induces localized stress concentration, whereas laminoplasty preserves a more uniform ligament loading pattern.

### Laminectomy significantly increases screw stress and overall mobility, while laminoplasty maintains postoperative stability

**FIGURE 6:**
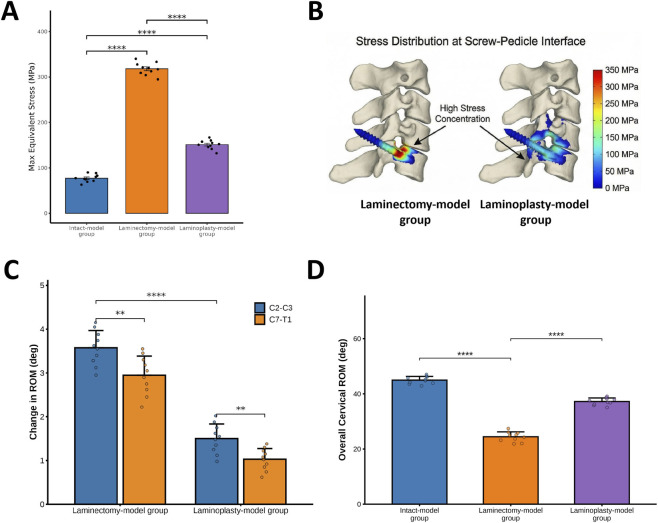
Comparison of screw stress distribution and postoperative stability trends. Note: **(A)** FEA-based comparison of the maximum equivalent stress at the C5-C6 cervical pedicle screw interface under intact, laminectomy, and laminoplasty conditions. **(B)** Stress contour maps illustrating the spatial distribution of stress at the screw-pedicle junction in the C5-C6 cervical pedicle screw fixation model (lateral view) following laminectomy and laminoplasty. **(C)** Biomechanical analysis comparing postoperative changes in ROM at adjacent segments (C2-C3 and C7-T1) under different surgical conditions. **(D)** Biomechanical analysis comparing the global cervical ROM among the intact, laminectomy, and laminoplasty models. Data are presented as mean ± SD, N = 10; ***p < 0.001; ****p < 0.0001.

Implant-related stress was analyzed to determine whether procedure-dependent load redistribution also translated into different fixation demands and implant-related mechanical risk. Under standardized physiological loading conditions (±1.0 N m), a C5–C6 cervical pedicle screw fixation model was used to systematically evaluate the effects of different surgical procedures on stress distribution within the internal fixation system and on overall postoperative stability. The results showed that the maximum equivalent stress at the screw–pedicle interface increased markedly in the laminectomy model and was substantially higher than that in the laminoplasty model (Figure 6A), indicating greater localized mechanical loading at the fixation interface after laminectomy.

Stress contour analysis further revealed distinct spatial patterns between the two surgical models. In the laminoplasty model, stress distribution along the pedicle screws was relatively uniform and dispersed, with stress mainly concentrated in the transition region between the screw shaft and pedicle bone. In contrast, the laminectomy model showed pronounced focal stress concentration at the junction between the screw tail and the pedicle interface (Figure 6B).

To evaluate the influence of surgical procedures on cervical stability, postoperative ROM at adjacent segments (C2–C3 and C7–T1) was also analyzed. The laminectomy model demonstrated a marked increase in adjacent segment ROM, whereas the laminoplasty model exhibited relatively smaller ROM changes (Figure 6C). Comparison of overall cervical ROM across the three models further demonstrated that the laminoplasty model showed the smallest change in global cervical mobility (Figure 6D), indicating better preservation of postoperative stability.

Overall, these findings indicate that laminectomy increases mechanical loading at the screw–pedicle interface and promotes greater postoperative cervical mobility, whereas laminoplasty preserves a more uniform stress distribution within the fixation system and better maintains cervical stability.

### Laminectomy induces excessive compensatory motion and stress concentration in adjacent segments, while laminoplasty maintains mechanical stability and prevents degeneration

**FIGURE 7:**
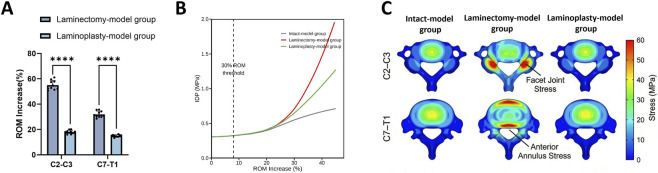
Analysis of postoperative biomechanical changes in adjacent segments. Note: **(A)** Quantitative ROM measurements were performed to analyze postoperative changes in mobility at the adjacent segments (C2-C3 and C7-T1) following the two surgical procedures (the laminectomy model and the laminoplasty model); **(B)** Based on nonlinear FEA, changes in IDP within the nucleus pulposus were evaluated after the ROM threshold exceeded 30%; **(C)** FEA stress contour maps illustrating the spatial distribution patterns of stress in adjacent segments under different surgical conditions. N = 10; ****p < 0.0001 between groups.

Under standardized physiological loading conditions (±1.0 N m), postoperative biomechanical responses in the adjacent cervical segments were quantitatively evaluated. Compared with the intact model, the laminectomy model showed a marked increase in adjacent-segment mobility, with significantly elevated ROM at both C2–C3 and C7–T1. These increases were substantially greater than those observed in the laminoplasty model (Figure 7A).

Nonlinear mechanical analysis further showed that once adjacent-segment ROM exceeded approximately 30% above the intact level, IDP and facet joint contact stress rose sharply relative to the intact-model condition. These increases were more pronounced in the laminectomy model than in the laminoplasty model (Figure 7B).

Stress contour maps revealed distinct differences in adjacent segment stress distribution between the two surgical models (Figure 7C). In the laminectomy model, high-stress regions were concentrated in the anterior annulus fibrosus of the inferior segment (C7–T1) and the medial facet region of the superior segment (C2–C3). In contrast, the laminoplasty model showed a more homogeneous stress distribution, with the pressure centroid remaining centrally located within the nucleus pulposus and without obvious peripheral stress concentration.

Overall, these results demonstrate that laminectomy increases compensatory motion and stress concentration in adjacent segments, whereas laminoplasty maintains a more stable mechanical environment in adjacent levels.

### MRI and FEA jointly reveal stress concentration and degenerative signal colocalization of the LF induced by laminectomy

MRI-based validation was then performed to determine whether the regions predicted to undergo stress concentration in the FE model corresponded to clinically observable early degenerative changes. To assess tissue-level degeneration and its biomechanical basis after different laminar decompression procedures, MRI parameters were integrated with FE predictions to analyze morphological alterations of the LF and their mechanical correlates.

**FIGURE 8:**
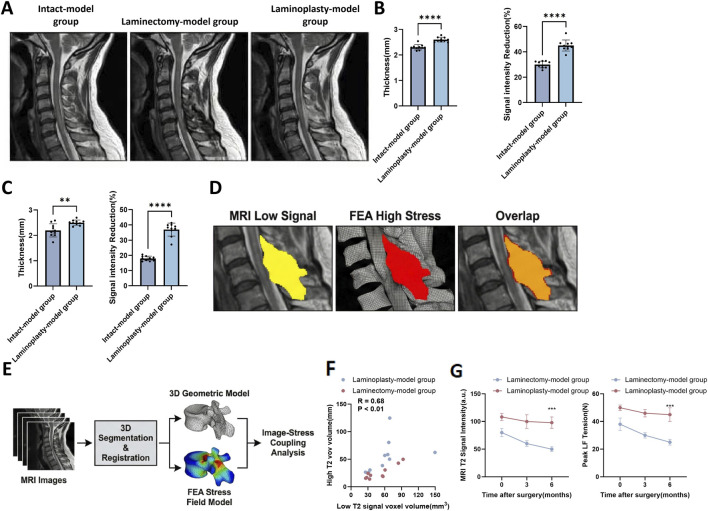
Consistency analysis between MRI imaging and FE modeling in postoperative LF stress alterations. Note: **(A)** T2-weighted sagittal MRI images showing morphological changes in the LF before and after laminectomy and laminoplasty; **(B,C)** Comparison of LF thickness and percentage of T2 signal attenuation across surgical groups; **(D)** Spatial overlap analysis between MRI low-signal regions and high-stress areas predicted by FE modeling, demonstrating correspondence between mechanical stress reconstruction and degenerative imaging features; **(E)** Schematic representation of three-dimensional geometric and stress field model construction based on MRI registration; **(F)** Correlation analysis between low-T2 signal voxels and high-stress voxel volumes; **(G)** Dynamic MRI signal tracking compared with FE tension trends, with parallel line graphs illustrating the time-dependent coupling between signal intensity and peak stress at 0, 3, and 6 months postoperatively. ** indicates *p <* 0.01; ***p < 0.001; ****p < 0.0001 between groups.

Postoperative sagittal T2-weighted MRI showed that, in the laminectomy-model group, the LF exhibited increased thickness, reduced signal intensity, and patchy low-signal regions. In contrast, the laminoplasty group showed only mild morphological changes, with relative preservation of internal signal characteristics (Figures 8A–C).

To evaluate the spatial correspondence between imaging findings and mechanical predictions, a registration-based analysis was performed to align regions of LF signal attenuation on MRI with areas of elevated equivalent stress identified by the FE model (Figure 8D). Using a 3D image registration template (Figure 8E) and voxel-wise overlap quantification, the spatial correlation between stress concentration regions and MRI low-signal zones reached r = 0.68 (*p <* 0.01) (Figure 8F).

Dynamic MRI follow-up further demonstrated temporal signal changes at 0, 3, and 6 months after surgery. The laminectomy-model group showed progressive reductions in T2 signal intensity that spatially corresponded to regions of elevated LF stress predicted by the FE model, whereas the laminoplasty group showed minimal temporal signal change (Figure 8G).

Overall, the FE model successfully identified postoperative stress redistribution patterns within the LF that corresponded spatially with degenerative imaging features observed on MRI.

### Laminectomy significantly increases screw fatigue stress and crack propagation, while laminoplasty enhances fixation stability and reduces loosening risk

Under multiaxial cyclic fatigue loading conditions (±1.0 N m, 10^5^ cycles), the mechanical behavior of the screw–bone interface in the posterior fixation system was evaluated for different decompression procedures. The analysis focused on stress distribution, fatigue crack propagation, and hysteresis response.

**FIGURE 9:**
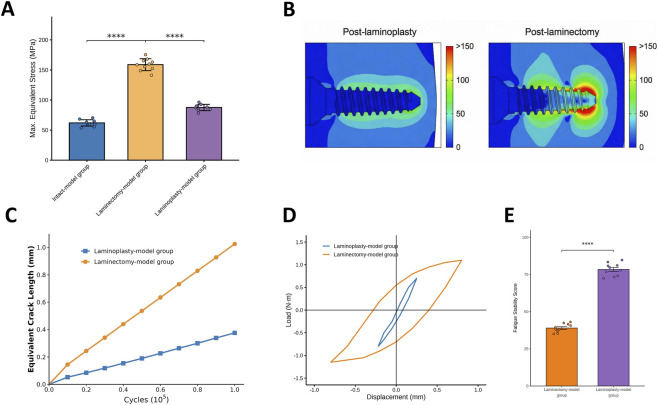
Comparison of screw fatigue stress response and loosening risk. Note: **(A)** FEA-derived maximum equivalent stress at the screw-bone interface under different surgical conditions. **(B)** Equivalent stress contour maps of the screw-bone interface, demonstrating localized stress concentration under laminectomy conditions. **(C)** Simulated evolution of equivalent crack length at the screw-bone interface under cyclic loading based on the Paris crack propagation model. **(D)** Loading-unloading hysteresis curves illustrating the interfacial response characteristics of the screw-bone interface under different surgical conditions. **(E)** Comparison of fatigue stability scores constructed by integrating multiple fatigue-related indicators. N = 10; **** indicates between-group comparison, *p <* 0.0001.

The maximum equivalent stress at the screw–bone interface increased markedly in the laminectomy model compared with the intact condition and showed a pronounced focal stress concentration pattern (Figure 9A). Stress contour maps further demonstrated that high-stress regions were mainly localized to the transition zone between the screw tail and the surrounding bone interface (Figure 9B).

Fatigue simulations based on the Paris crack propagation model showed that, under identical cyclic loading, the equivalent crack length at the screw–bone interface increased more substantially in the laminectomy model, indicating a higher crack growth rate than in the laminoplasty model (Figure 9C). In contrast, the laminoplasty model exhibited a more uniform peri-screw stress distribution and lower peak stress levels.

Analysis of loading–unloading hysteresis behavior further revealed differences in fixation stability. The laminectomy model showed larger hysteresis loop areas and greater residual displacement at the screw–bone interface, indicating increased inelastic deformation and interfacial instability. By contrast, the laminoplasty model exhibited smaller hysteresis loops and minimal residual displacement across loading cycles (Figure 9D).

To comprehensively assess fixation stability, multiple mechanical indicators, including maximum equivalent stress, crack propagation rate, hysteresis response, and residual displacement, were integrated into a multidimensional fatigue stability score. The laminoplasty model showed a significantly higher overall fatigue stability score than the laminectomy model (*p <* 0.001) (Figure 9E). Overall, these results demonstrate that laminectomy increases fatigue stress and crack propagation at the screw–bone interface, whereas laminoplasty maintains a more stable mechanical environment within the fixation system.

### Construction of a multidimensional biomechanical degeneration scoring system

**FIGURE 10:**
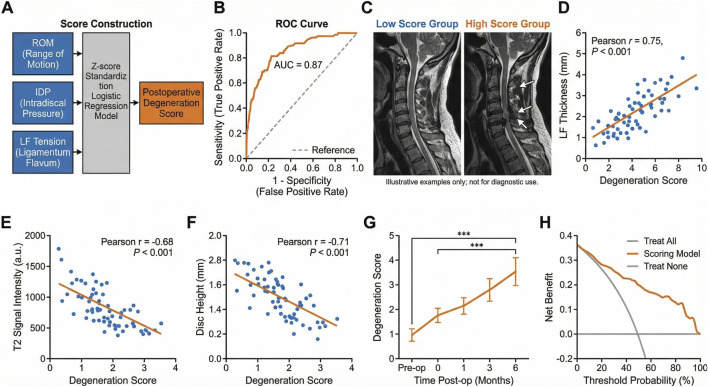
Construction and validation of a multidimensional postoperative degeneration scoring system. Note: **(A)** Schematic workflow of the multidimensional postoperative degeneration scoring system constructed based on segmental ROM, IDP, and LF tension. **(B)** Receiver operating characteristic (ROC) curve evaluating the discriminative performance of the scoring model. **(C)** Representative postoperative sagittal T2-weighted MRI images illustrating imaging differences between the high-score and low-score groups, presented for visual demonstration of score-based stratification rather than for segmental or anatomical determination. **(D)** Pearson correlation analysis between LF thickness and the degeneration score. **(E)** Pearson correlation analysis between T2 signal intensity and the degeneration score. **(F)** Pearson correlation analysis between intervertebral disc height and the degeneration score. **(G)** Temporal trends of degeneration scores at different postoperative time points. **(H)** DCA comparing the net clinical benefit of the degeneration scoring model with Treat-all and Treat-none strategies. N = 10; *** indicates between-group comparison, *p <* 0.001.

Finally, the identified biomechanical indicators were integrated into a scoring framework to determine whether the observed mechanical cascade could be translated into a clinically useful tool for postoperative risk stratification. To systematically quantify the combined effects of different decompression procedures on the risk of postoperative ASD, a multidimensional degeneration risk model was developed by integrating three key biomechanical parameters: segmental ROM, IDP, and LF tension (Figure 10A). Before model construction, principal component analysis (PCA) was performed to reduce feature dimensionality and visualize the distribution of biomechanical variables. After Z-score normalization, all variables were entered into a logistic regression model. Cervical segments derived from the FE simulations were treated as independent samples. During dataset partitioning, segments from the same participant were retained within the same split to avoid data leakage. Approximately 80% of the samples were used for model training, and the remaining 20% were reserved for independent testing. Model training used standard hyperparameter settings with a maximum of 1,000 iterations, and logistic regression was implemented with L2 regularization (ridge penalty) to reduce overfitting. Fivefold cross-validation was performed to evaluate model stability. Receiver operating characteristic (ROC) analysis yielded an area under the curve (AUC) of 0.87, indicating good predictive performance of the scoring system (Figure 10B).

Based on the median score, simulated samples were divided into high-score and low-score groups. Representative postoperative sagittal T2-weighted MRI images were selected to illustrate the imaging features of the two groups (Figure 10C). The high-score group more frequently showed LF thickening and reduced signal intensity, whereas imaging changes in the low-score group were relatively mild.

Pearson correlation analysis was then performed between degeneration scores and objective imaging parameters extracted from postoperative MRI, including LF thickness, T2 signal intensity, and intervertebral disc height. The degeneration score showed a significant positive correlation with LF thickness and significant negative correlations with T2 signal intensity and disc height (Figures 10D–F).

Temporal analysis further showed that the degeneration score changed progressively during the 0–6 months postoperative follow-up period, consistent with the direction of intervertebral load redistribution and postoperative biomechanical adaptation (Figure 10G).

The clinical utility of the scoring system was further evaluated using decision curve analysis (DCA). The proposed model demonstrated greater net clinical benefit than both the treat-all and treat-none strategies across a wide range of threshold probabilities (Figure 10H), indicating potential value for postoperative risk stratification and follow-up assessment.

## Discussion

This study investigated the long-term biomechanical differences between two widely used posterior cervical decompression procedures, laminoplasty and laminectomy, using multiscale three-dimensional FEA. Posterior cervical decompression is commonly performed for the treatment of spinal cord compression disorders, yet the postoperative biomechanical consequences of different decompression strategies remain incompletely understood (Han et al., 2025). Laminoplasty preserves part of the posterior column and may contribute to maintenance of spinal stability (Yu W. et al., 2024). In contrast, laminectomy achieves more extensive neural decompression by completely removing the laminar structures within the surgical region (Fehlings et al., 2017). These structural differences may lead to distinct long-term effects on spinal function and adjacent segment integrity (Liu et al., 2013). In this study, FEA was used to comprehensively compare the long-term mechanical performance of the two procedures, with a particular focus on stress distribution under dynamic loading, modulation of LF tension, and the stability of screw fixation systems, thereby enabling a holistic biomechanical assessment of both surgical approaches.

Validation of the FE model is a critical component of the study. High-resolution geometric reconstruction and carefully defined material properties were applied to reproduce the anisotropic behavior and dynamic mechanical responses of cervical tissues with high fidelity. Comparison of simulation outputs with previously reported cadaveric experimental data showed relative errors of less than ±10%, indicating strong agreement with published findings. Notably, the predicted patterns of facet joint contact stress and IDP under dynamic loading closely paralleled experimental observations. Compared with previous studies, this work further integrated high-resolution imaging data, MRI-based LF assessment, and postoperative adjacent segment motion-sequence analysis, allowing more detailed characterization of postoperative dynamic biomechanical mechanisms. Collectively, these results support the validity of the model and suggest that it may serve as a scalable quantitative platform for predicting postoperative biomechanical outcomes.

Based on the FEA results, laminoplasty was associated with a smaller postoperative increase in overall cervical ROM, thereby better preserving global spinal stability. This advantage was observed across multiple motion patterns, including flexion, extension, and axial rotation, suggesting that laminoplasty may provide superior long-term biomechanical stability after surgery. In contrast, although laminectomy achieved more extensive decompression, it markedly increased ROM at both the operative and adjacent segments, resulting in stress concentration that may accelerate the development of ASD. Notably, distinct stress peaks were observed in the facet joint regions following laminectomy, suggesting that stress redistribution may represent a key mechanism underlying joint degeneration and secondary symptom development. By comparing the mechanical characteristics of the two procedures, this study demonstrates that laminoplasty offers advantages in preserving postoperative motion balance and reducing stress concentration, thereby supporting its potential long-term clinical benefits.

Taken together, these findings support a coherent mechanistic pathway rather than a series of isolated biomechanical observations. Relative preservation of the posterior elements after laminoplasty limited the postoperative increase in ROM, which in turn reduced compensatory load redistribution to the facet joints, intervertebral discs, and LF. This more balanced mechanical environment was accompanied by lower implant-related stress and less pronounced degeneration-related changes on MRI. In contrast, more extensive posterior structural removal after laminectomy was associated with greater postoperative mobility, focal stress concentration in posterior and adjacent-segment structures, and a stronger correspondence with early degenerative imaging manifestations. Accordingly, the degeneration scoring model should be interpreted as a downstream integration of these interrelated mechanical changes rather than as an independent analytical module.

This study further established a postoperative degeneration risk prediction model based on FEA by integrating multiple biomechanical indicators, including IDP, facet joint stress, and LF tension, into a unified analytical framework. This multidimensional biomechanical model not only deepens mechanistic understanding of postoperative degeneration but also provides quantitative support for individualized treatment planning. Notably, laminoplasty significantly attenuated postoperative increases in adjacent-segment IDP, underscoring its potential biomechanical advantage in reducing degenerative loading and supporting its consideration in surgical decision-making. Validation against postoperative MRI-based degeneration grading further confirmed the predictive performance and clinical relevance of the model. With larger cohorts and future multicenter external validation, this scoring system may facilitate postoperative risk stratification and personalized follow-up management in cervical spine surgery, thereby contributing to the advancement of precision spinal care.

Overall, this study systematically compared the biomechanical effects of two major posterior cervical decompression procedures using a high-resolution FE model. The findings indicate that laminoplasty offers clearer advantages in preserving spinal stability, optimizing stress distribution, and reducing mechanical loading in adjacent segments, whereas laminectomy is more likely to increase overall cervical mobility and induce localized stress concentration, thereby potentially increasing the risk of postoperative degeneration. Several limitations should nevertheless be acknowledged. First, the sample size was relatively modest and derived from a single-center cohort, which may limit the generalizability of the findings. Second, the three model groups were constructed from independent individuals rather than from longitudinal pre- and postoperative data from the same subjects; accordingly, the present results mainly reflect group-level biomechanical trends rather than within-subject temporal changes. Third, muscular effects were represented indirectly through follower loading and ligamentous constraints rather than through patient-specific active muscle modeling, which may underestimate the complexity of physiological dynamic loading. Fourth, to balance numerical stability and computational efficiency, several constitutive and interface conditions, including intervertebral disc material behavior, joint contact definitions, and the screw–bone interface, were simplified; therefore, local stress peaks and fatigue-related responses should be interpreted with caution. Fifth, MRI follow-up was limited mainly to early postoperative time points, and the proposed degeneration scoring system has not yet been externally validated in an independent cohort. Its long-term predictive performance and clinical applicability remain to be established. Future studies should address these limitations through multicenter, large-scale, and longitudinal validation; incorporation of patient-specific muscle-driven loading conditions; refinement of material and contact models; and integration of longer-term and higher-dimensional imaging modalities, such as four-dimensional MRI, to further improve model fidelity, robustness, and translational value. Taken together, these findings provide a meaningful biomechanical basis for postoperative risk prediction and individualized surgical planning and further support the development of precision-medicine strategies for complex cervical spine surgery.

## Data Availability

The original contributions presented in the study are included in the article/Supplementary Material, further inquiries can be directed to the corresponding author.

## Supplementary material

SUPPLEMENTARY FIGURE S1 Workflow of subject selection, imaging acquisition, and data processing. Note: This diagram illustrates the complete workflow for subject selection and imaging data processing. A total of 78 healthy volunteer images were retrieved from the clinical imaging database. After screening based on imaging quality and anatomical criteria, 30 eligible subjects were included. The participants were categorized into three simulation groups—the intact-model group, the laminectomy-model group, and the laminoplasty-model group—and underwent standardized CT and MRI scanning. All images were anonymized and format-converted before being used for FE geometric modeling of the C3-C7 segments and subsequent biomechanical analysis.

SUPPLEMENTARY FIGURE S2 Workflow of *in vitro* FE modeling and mechanical loading. Note: This figure presents the workflow for *in vitro* FE modeling and mechanical loading. High-resolution MRI and CT images were segmented and reconstructed in Mimics software, optimized in Geomagic Studio, and then meshed in HyperMesh, with anisotropic material properties assigned based on literature data. The model was subjected to physiological loading conditions, including flexion, extension, lateral bending, and axial rotation, within ANSYS Workbench. Key mechanical parameters—such as ROM, IDP, and stress distribution—were extracted. This workflow ensured both anatomical fidelity and computational precision, providing a robust foundation for subsequent surgical simulations and validation.

SUPPLEMENTARY FIGURE S3 Technical roadmap of cervical spine surgical modeling, loading setup, and ASD simulation. Note: This figure illustrates the overall workflow of FEA for cervical spine surgical modeling. Beginning with imaging-based reconstruction and surgical geometric modeling (Step 1), the process proceeds to the definition of mechanical loading and boundary constraints (Step 2). Subsequently, under multiple motion modes, the stability and stress distribution of the fixed segments and screw interfaces are analyzed (Step 3). The mechanical behavior of adjacent segments under degenerative conditions is then simulated (Step 4), followed by visualization and quantitative prediction of postoperative stability and degeneration risk (Step 5). This technical framework provides a theoretical basis and decision-making support for optimizing intraoperative decompression range and assessing postoperative degeneration risk.
