# Supplementary figures and images for: Individualized finite element simulation integrating high-resolution magnetic resonance imaging and clinical imaging validation elucidates the effects of laminoplasty and laminectomy on postoperative mechanical stability and degeneration risk
Source: Front Bioeng Biotechnol. 2026 Jul 14;14:1763758. doi: 10.3389/fbioe.2026.1763758 (PMC13408222; doi:10.3389/fbioe.2026.1763758)

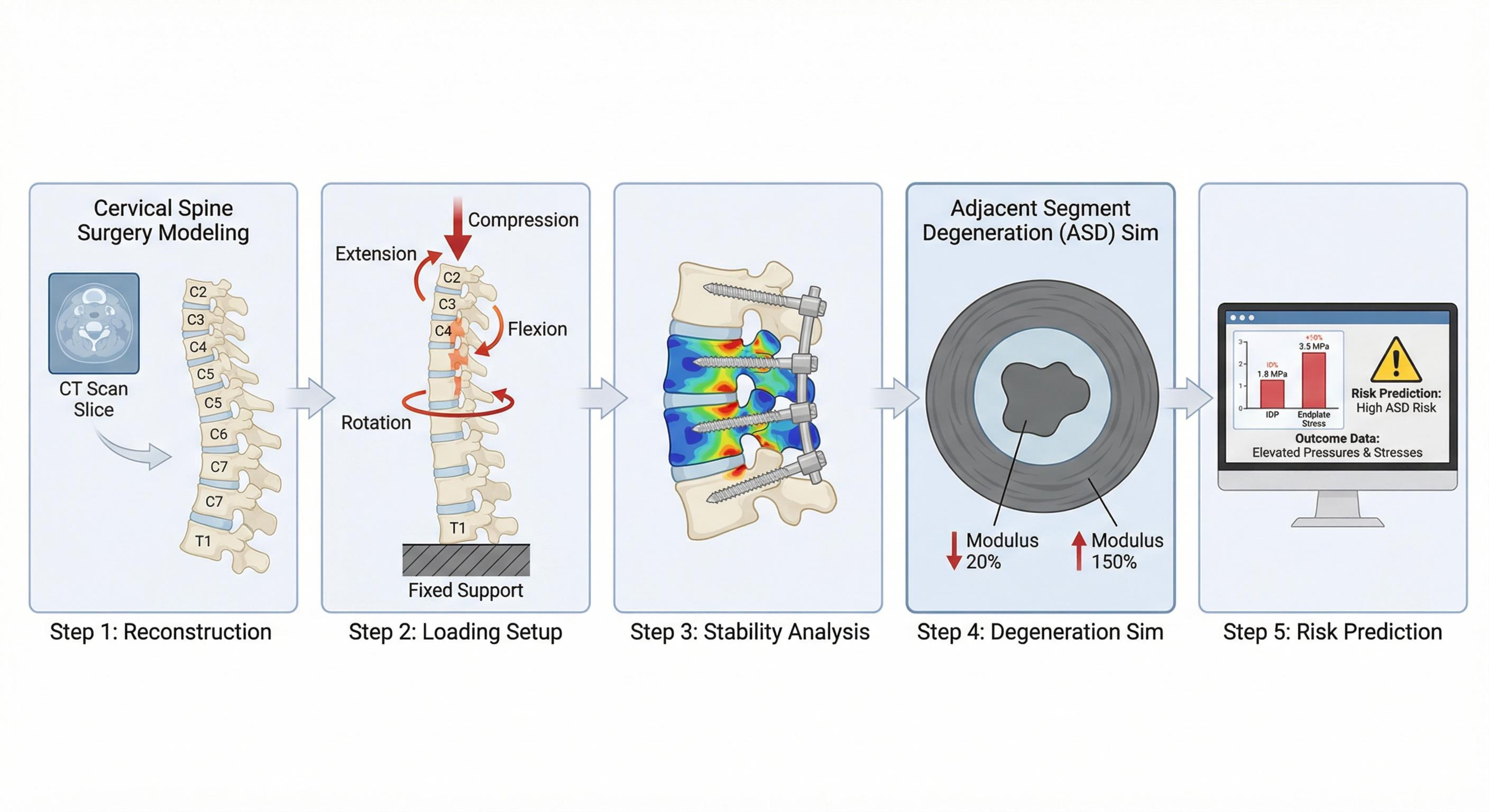

Supplement: Supplementary file 1 [file Image3.jpeg]

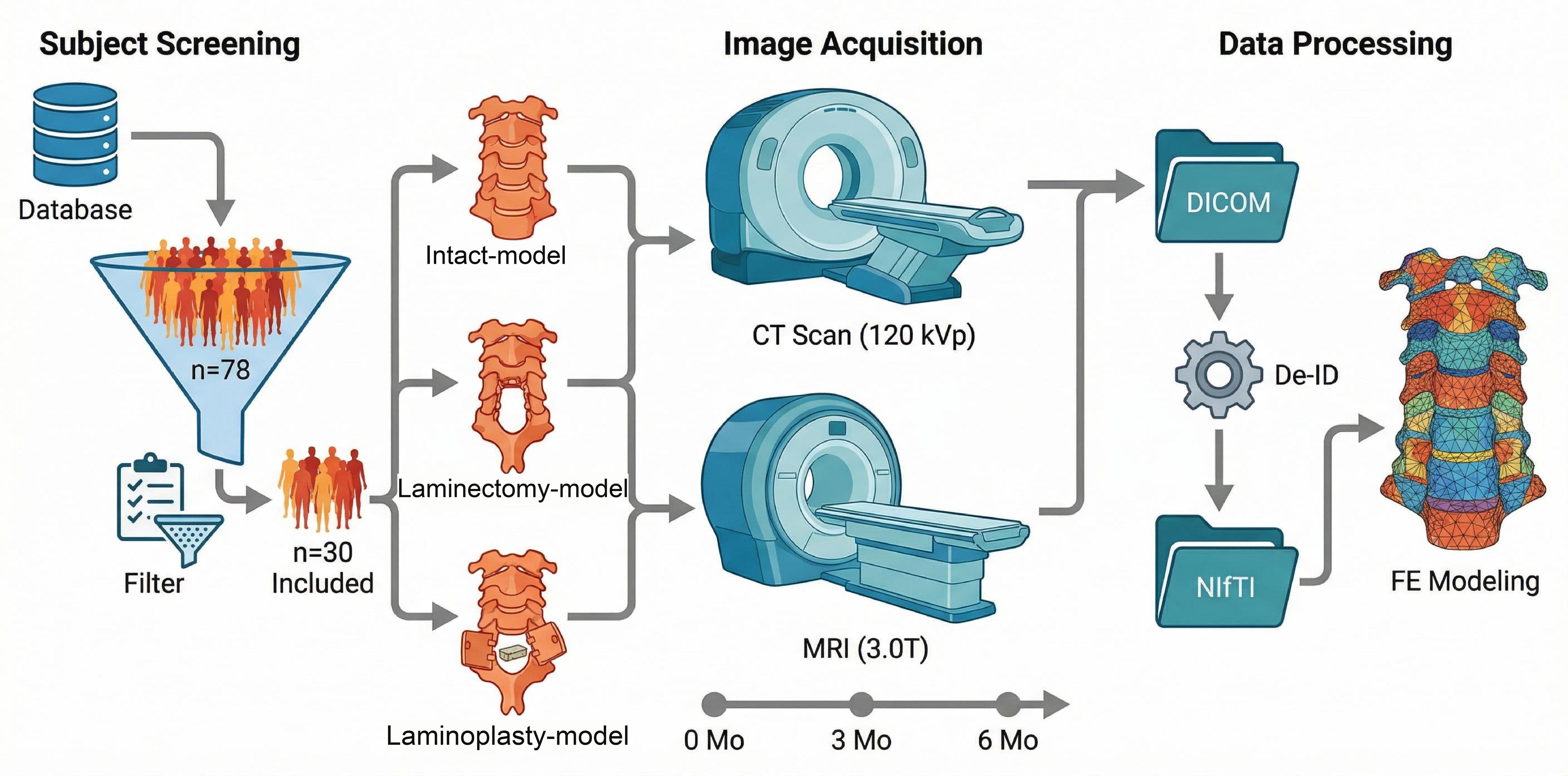

Supplement: Supplementary file 3 [file Image1.jpeg]

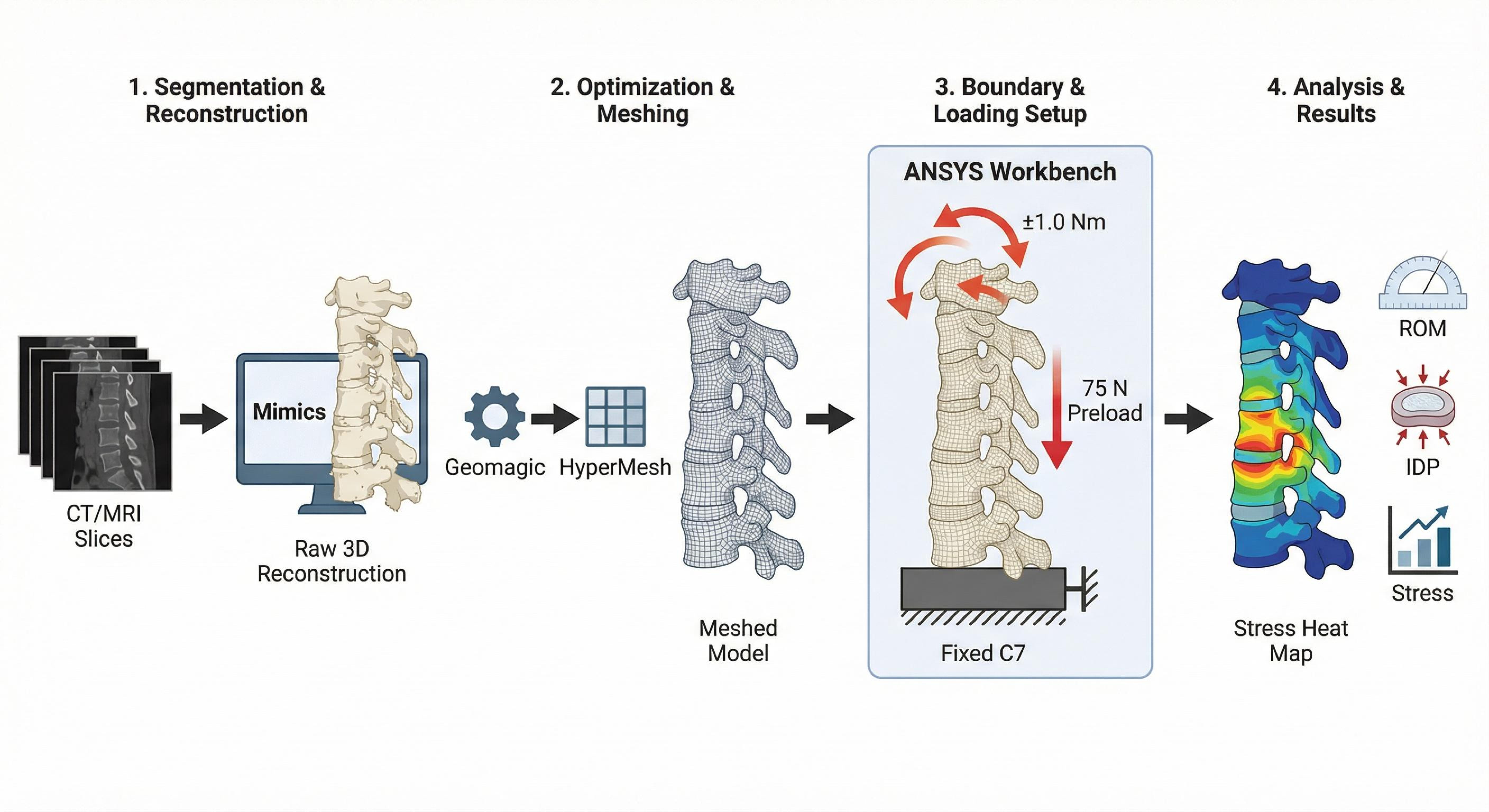

Supplement: Supplementary file 4 [file Image2.jpeg]
