# Supplementary material for: Individualized finite element simulation integrating high-resolution magnetic resonance imaging and clinical imaging validation elucidates the effects of laminoplasty and laminectomy on postoperative mechanical stability and degeneration risk
Source: Front Bioeng Biotechnol. 2026 Jul 14;14:1763758. doi: 10.3389/fbioe.2026.1763758 (PMC13408222; doi:10.3389/fbioe.2026.1763758)
Supplement: Supplementary file 2 [file Table1.docx]

**Table S1.** Constitutive models, material parameters, and contact/interface definitions used in the cervical finite element model.

| **Component** | **Material model** | **Specific parameter values** | **Supporting references** |
| --- | --- | --- | --- |
| Cortical bone | Linear elastic | Elastic modulus = 12,000 MPa; Poisson’s ratio = 0.30 | PMID: 31035068; PMID: 33142209 |
| Cancellous bone | Linear elastic | Elastic modulus = 100 MPa; Poisson’s ratio = 0.20 | PMID: 31035068; PMID: 33142209 |
| Nucleus pulposus | Neo-Hookean, nearly incompressible | C10 = 0.12 MPa; D1 = 0.001 MPa⁻¹; in ASD simulation, the effective elastic modulus was reduced to 20% of baseline | PMID: 31035068; PMID: 33142209; PMID: 16424844; PMID: 21244955 |
| Annulus fibrosus matrix | Hyperelastic matrix + fiber reinforcement | Ground-matrix elastic modulus = 4.2 MPa; Poisson’s ratio = 0.45; fiber orientation = ±30° | PMID: 31035068; PMID: 33142209 |
| Annulus fibrosus fibers | Tension-only fiber-reinforced | Fiber elastic modulus = 550 MPa; Poisson’s ratio = 0.30; in ASD simulation, annulus effective modulus was increased to 150% of baseline | PMID: 31035068; PMID: 33142209; PMID: 16424844; PMID: 21244955 |
| Ligaments (ALL, PLL, LF, ISL, CL) | Nonlinear tension-only | Nonlinear stiffness assigned according to previously validated cervical FE models; tension-only behavior used for all ligaments | PMID: 39395967; PMID: 31092257 |
| Facet cartilage / facet contact | Linear elastic + surface-to-surface contact | Elastic modulus = 10 MPa; Poisson’s ratio = 0.40; tangential friction coefficient = 0.10 | PMID: 24848166; PMID: 31035068 |
| Titanium screws / fixation devices | Linear elastic | Elastic modulus = 110,000 MPa; Poisson’s ratio = 0.30; screw diameter = 3.5 mm; screw length = 14–16 mm | PMID: 14560082; PMID: 37029671; PMID: 35482019; PMID: 37062984 |
